# Supplementary material for: Negative health impacts of navigating the healthcare system for musculoskeletal conditions: A scoping review protocol
Source: PLoS One. 2024 Oct 25;19(10):e0311970. doi: 10.1371/journal.pone.0311970 (PMC11508056; doi:10.1371/journal.pone.0311970)
Supplement: Attachment — Submitted filename: PONE-D-24-26506_Response to Reviewers.docx [file pone.0311970.s002.docx]

TOPIC: Review response letter

MANUSCRIPT ID: PONE-D-24-26506

Dear Dr Amin Nakhostin-Ansari and Editorial Team:

As per your instructions, received by email on August 15, 2024, this is our response letter accompanying the submission of manuscript (PONE-D-24-26506) to *PLOS One*. Below we provide a response to each point raised by the two reviewers from *PLOS One*, with reference to where changes can be viewed in the submitted manuscript.

We thank the reviewers for their helpful review and comments and have detailed the changes made in the submitted manuscript.

**Editor Comment 1:** Please ensure that your manuscript meets PLOS ONE's style requirements, including those for file naming. The PLOS ONE style templates can be found at 
<https://journals.plos.org/plosone/s/file?id=wjVg/PLOSOne_formatting_sample_main_body.pdf> and 
<https://journals.plos.org/plosone/s/file?id=ba62/PLOSOne_formatting_sample_title_authors_affiliations.pdf>

**Author Response 1:** We have adhered to the style requirements to the best of our knowledge.

**Editor Comment 2:** Please provide a complete Data Availability Statement in the submission form, ensuring you include all necessary access information or a reason for why you are unable to make your data freely accessible. If your research concerns only data provided within your submission, please write "All data are in the manuscript and/or supporting information files" as your Data Availability Statement.

**Author Response 2:** We have updated our data availability statement in the submission site to state:

"All data are in the manuscript and/or supporting information files"

**Reviewer 1 Comment 1:** Excellent concept and research question. The methodology has been well thought of. A couple of main things to address:

**Author Response 1:** We thank the reviewer for their encouraging comment and for taking the time to provide a review of our manuscript.

**Reviewer 1 Comment 2**: Settings was not well described and no sub-group analysis planned. Is this specific to some countires or region? The perception or consequences of navigating health system will be different for different countries. So analysis needs to be done with this lens.

**Author Response 2**: We have included a new category in our extraction template to chart each paper based on study setting (e.g., acute, primary, community) and location of study (e.g., region, country). These changes are located on page 15, Table 2, lines 152-153 and page 16, line 159.

**Reviewer 1 Comment 3:** The search strategy is not very comprehensisve and may not pick up many MSK issues. This needs looking into.

**Author Response 3**: Thank you for your comment and the opportunity to further explain our search strategy. We purposively selected the most common MSK conditions (low back, knee, shoulder) and those that typically require healthcare system intervention. Additionally, these three MSK conditions have been identified as priority conditions in Alberta, Canada as this scoping review will inform current initiatives of the MSK-Transformation Project of Alberta Health Services in Canada. We worked with a trained research librarian to craft the search strategy and piloted the strategy to ensure that exemplar papers we previously identified were captured in the search. Although our strategy will not capture all MSK conditions (e.g., hand, hip) we do believe it is comprehensive enough to capture papers examining the focal MSK conditions (i.e., knee, low back, shoulder).

We have revised the methods (Page 6-7, lines 115-119) and limitation section (Page 17, lines 189-192) to provide more details on the search strategy and acknowledge that our strategy is not intended to capture all MSK conditions.

**Reviewer 2 Comment 1:** Thanks for the protocol. This looks a well thought out and detailed study protocol.

**Author Response 1**: We thank the reviewer for their kind comment and thoughtful review.

**Reviewer 2 Comment 2:** Is this study looking at qualitative or quantitative papers? I’m assuming both but it might be good to explicitly state this.

**Author Response 2**: The reviewer is correct; this study is reviewing both qualitative and quantitative papers. We have clarified this point in text (Page 6, lines 102-103).

**Reviewer 2 Comment 3:** Do you have plans for regional i.e. country specific analysis? I’d be tempted to add location of cohort to your extraction sheet. You may find very interesting differences between different countries and regions due to differing social contexts.

**Author Response 3**: We have included a new category in our extraction template to chart each paper based on study setting (e.g., acute, primary, community) and location of study (e.g., region, country). This change is located on page 15, Table 2, lines 152-153 and page 16, line 159.

**Reviewer 2 Comment 4:** The search strategy limits articles to adult populations, but I don’t think this is detailed in inclusion/exclusion criteria. What age group are you looking at?

**Author Response 4**: We are looking at adult populations (i.e., 18 or older). We have revised the inclusion criteria to include this criterion (Page 5, lines 83-84).

**Reviewer 2 Comment 5:** Inclusion criteria 2nd para, models of health. Is this part of the inclusion criteria? It seems a little disjointed. If so I think the paper should state something like ‘the health impact described must fit within one of eight dimensions’ or ‘we define health impact as’

**Author Response 5**: We have revised the second paragraph to incorporate the reviewer’s suggested language (Page 5, lines 90-91).

**Reviewer 2 Comment 6:** Will you be searching the ref lists of included papers? i.e. backwards and forwards searching. Will any papers be included by expert recommendation etc?

**Author Response 6**: We will be searching the reference lists for included papers (i.e., citation chaining), we have now included this statement in the methods section (Page 13, lines 140-146).

Prior to connecting with the research librarian, our team (which included bone and joint health experts) assembled a small number of exemplary papers which met the inclusion criteria. We tested the accuracy of the search strategy by checking if the exemplary papers were included within the results from the search. If not, the strategy was revised. We have now included some text in the methods section explaining this additional step (Page 6-7, lines 115-119).

**Reviewer 2 Comment 7:** Are you including people seeking medical help for traumatic injury? Your paper seems written up in the context of longer term conditions but if a study looks at emergency admissions for knee trauma or sports injury would that be included?

**Author Response 7**: Yes, we are including both chronic and acute MSK conditions. We have revised the manuscript to include this detail (Page 6, line 109).

**Reviewer 2 Comment 8:** How are you dealing with more generalised conditions, such as patient seeking help for general MSK pain (most of which will fall into knee, low back and shoulder)? Are these excluded, if so it may be useful to specify that papers exploring generalised conditions which may include knee, low back and shoulder are excluded)

**Author Response 8**: Generalized MSK pain is included in our search strategy. The rationale for this is that MSK pain is typically the reason individuals will seek healthcare and thus have to navigate the healthcare system. Many of these individuals who seek help for pain do go on to be diagnosed with an MSK condition. We have revised the manuscript to include generalized pain as part of the search (Page 6, line 110-111).

**Reviewer 2 Comment 9:** The first sentence of the discussion seems overly complex, I think it may be better as two sentences, Also are resources narrowing everywhere? If it’s a global study that assumption may not be true everywhere.

**Author Response 9**: We have revised the first sentence of the discussion to split it into two sentences (Pages 16-17, lines 172-177). In addition, we have provided two citations to support the narrowing of healthcare services for chronic (including MSK) conditions globally. First, we cite a WHO press release that discusses the progress of one of the World Health Organization Sustainable Development Goals: the expansion of essential health services. In this article the authors report that since 2015 there have been no improvements in access to health services for non-communicable diseases and this is in the face of increasing global populations. Second, we cite an article examining unmet need for rehabilitation using data from the Global Burden of Disease project, this article finds that need for rehabilitations has increased by 63% from 1990 to 2019 internationally.

We again thank the editor and reviewers for their comments, and we hope these revisions are satisfactory.
