# Supplementary material for: Negative health impacts of navigating the healthcare system for musculoskeletal conditions: A scoping review protocol
Source: PLoS One. 2024 Oct 25;19(10):e0311970. doi: 10.1371/journal.pone.0311970 (PMC11508056; doi:10.1371/journal.pone.0311970)
Supplement: Attachment — Submitted filename: Revisions.docx [file pone.0311970.s003.docx]

I have reviewed the manuscript with great interest and enthusiasm. The significance of the title is noteworthy, and the manuscript is presented in an appropriate format. It would benefit from minor revisions to further enhance its clarity and impact. Upon addressing these revisions, the manuscript appears well-suited for publication in PLOS ONE.

- In the eligibility criteria, the manuscript mentions the **use of an eight-dimension health model** to define health impacts. While this is a solid approach, it would benefit from a **clear definition of what constitutes a health impact in each dimension** (e.g., what qualifies as a financial or environmental health impact). Include specific examples or parameters that would be categorized under each dimension of health (social, emotional, physical, etc.) to improve replicability. For instance, would loss of employment due to prolonged pain fall under financial health impacts? Clarifying these boundaries will enhance the scoping review's focus.
- The manuscript emphasizes that no geographical limits will be applied to the search strategy. This global approach is commendable, but there should be **consideration for regional variability in healthcare systems**. Consider adding an **analysis or categorization by country/region** to assess if the negative health impacts vary by healthcare system structures or if not applicable, authors should discuss it in limitations.
- The manuscript's search strategy focuses on the three most common MSK conditions (low back, knee, shoulder). While this focus is understandable, it might limit the scope of the review as **other MSK conditions,** such as hip and neck issues, are also prevalent and burdensome.
- Patients with MSK conditions often have comorbidities (e.g., obesity, cardiovascular issues) that might complicate their navigation through the healthcare system. It could be beneficial if authors clarify how the review will handle studies where comorbidities are present.
