# Supplementary material for: Negative health impacts of navigating the healthcare system for musculoskeletal conditions: A scoping review protocol
Source: PLoS One. 2024 Oct 25;19(10):e0311970. doi: 10.1371/journal.pone.0311970 (PMC11508056; doi:10.1371/journal.pone.0311970)
Supplement: Attachment — Submitted filename: PONE-D-24-26506 R2_Response to Reviewers.docx [file pone.0311970.s004.docx]

TOPIC: Review response letter

MANUSCRIPT ID: PONE-D-24-26506R1

Dear Dr Amin Nakhostin-Ansari and Editorial Team:

As per your instructions, received by email on September 13, 2024, this is our response letter accompanying the submission of manuscript (PONE-D-24-26506 R2) to *PLOS One*. Below we provide a response to each point raised by the two reviewers from *PLOS One*, with reference to where changes can be viewed in the submitted manuscript.

We thank the reviewers for their helpful review and comments and have detailed the changes made in the submitted manuscript.

**Reviewer 2 Comment 1:** Thanks for your response which answers all my queries. I believe the changes and clarifications make the paper clearer. Good luck with the study

**Author Response 1:** We thank the reviewer for their response and for taking the time to provide a review of our manuscript. Their suggestions contributed to a much stronger manuscript.

**Reviewer 3 Comment 1**: I have reviewed the manuscript with great interest and enthusiasm. The significance of the title is noteworthy, and the manuscript is presented in an appropriate format. It would benefit from minor revisions to further enhance its clarity and impact. Upon addressing these revisions, the manuscript appears well-suited for publication in PLOS ONE.

**Author Response 1**: We thank the reviewer for their encouraging comment and for taking the time to review our manuscript.

**Reviewer 3 Comment 2:** In the eligibility criteria, the manuscript mentions the use of an eight-dimension health model to define health impacts. While this is a solid approach, it would benefit from a clear definition of what constitutes a health impact in each dimension (e.g., what qualifies as a financial or environmental health impact). Include specific examples or parameters that would be categorized under each dimension of health (social, emotional, physical, etc.) to improve replicability. For instance, would loss of employment due to prolonged pain fall under financial health impacts? Clarifying these boundaries will enhance the scoping review's focus.

**Author Response 2**: Thank you for your comment and the clarify our chosen model of health. We have revised the manuscript to include definitions and examples of each of the eight dimensions of the health model in the newly include Table 1 on Page 5-6. Of note, upon reviewing our referred sources again, we opted to collapse financial health into a large group of “occupational health”. The reviewer’s example of loss of employment due to prolonged pain due to untreated MSK conditions would fall under occupational health impacts.

**Reviewer 3 Comment 3:** The manuscript emphasizes that no geographical limits will be applied to the search strategy. This global approach is commendable, but there should be consideration for regional variability in healthcare systems. Consider adding an analysis or categorization by country/region to assess if the negative health impacts vary by healthcare system structures or if not applicable, authors should discuss it in limitations.

**Author Response 3**: Thank you for this suggestion. We had previously included country/region in our extraction criteria. We have now revised our extraction criteria to include type of healthcare system (e.g., universal healthcare system, social health insurance, private health insurance, out-of-pocket system, mixed system, community-based or faith-based healthcare systems) (see Table 3, formerly Table 2 on page 15). In addition, we have clarified in our methods section our intention to conduct a stratified synthesis by type of health system to examine any differences in negative health impacts by healthcare system structure. This sentence on page 17 lines 170-173 now states, “In addition, given that we have not placed any geographic limits on this scoping review, we will examine whether there are any differences in negative health impacts, or causes of impacts, based on the type of healthcare system in place in the study setting.”

**Reviewer 3 Comment 4:** The manuscript's search strategy focuses on the three most common MSK conditions (low back, knee, shoulder). While this focus is understandable, it might limit the scope of the review as other MSK conditions, such as hip and neck issues, are also prevalent and burdensome.

**Author Response 4**: The reviewer is correct; this study is limited in its examination of conditions such as hip and neck issues. We purposively selected three of the most common MSK conditions (low back, knee, shoulder) and those that typically require healthcare system intervention. Additionally, these three MSK conditions have been identified as priority conditions in Alberta, Canada as this scoping review will inform current initiatives for the healthcare system in Alberta, Canada. Although our strategy will not capture all MSK conditions we do believe it is comprehensive enough to capture papers examining the focal MSK conditions (i.e., knee, low back, shoulder). We have recognized this limitation on page 18 lines 191-194.

**Reviewer 3 Comment 5:** Patients with MSK conditions often have comorbidities (e.g., obesity, cardiovascular issues) that might complicate their navigation through the healthcare system. It could be beneficial if authors clarify how the review will handle studies where comorbidities are present.

**Author Response 5**: The reviewer is correct, patients with MSK conditions often have comorbidities when seeking healthcare, therefore we have included an additional column in our extraction criteria on patient characteristics where these details can be extracted and summarized to provide important contextual information in the synthesis. We have revised the extraction table (Table 3, formerly Table 2 on page 15).

We again thank the editor and reviewers for their comments, and we hope these revisions are satisfactory.
